# Prospective Analysis of Lipid Variations in Hyperthyroid Subjects from Lahore, Pakistan

**DOI:** 10.1155/2021/9936782

**Published:** 2021-09-03

**Authors:** Muhammad Amir Iqbal, Shaaf Ahmad, Tamseela Mumtaz, Zahra Naseem, Javeria Malik, Husna Ahmad, Nabila Roohi

**Affiliations:** ^1^Institute of Zoology, University of the Punjab, Canal Road, Lahore, Punjab 54590, Pakistan; ^2^King Edward Medical University/Mayo Hospital, Hospital Road, Lahore, Punjab 54000, Pakistan; ^3^Department of Zoology, Government College for Women University, Faisalabad, Punjab, Pakistan

## Abstract

Perturbations in the actions of T_3_ and T_4_ influence the normal metabolic pathways. Responsiveness of lipid biomarkers like LDL-C, HDL-C, TC, TG, Apo-A, and Apo-B after rehabilitation of thyroid profile attaining euthyroid state was determined. A total of 179 age-matched subjects of both genders were recruited for this research. Sixty healthy controls, thirty-four subclinical, fifty overt hyperthyroid, and thirty-five follow-up subjects having 3 months of Carbimazole therapy were enrolled. Biochemical analysis was performed by chemistry analyzer, RIA, and ELISA. One-way ANOVA was applied for the statistical analysis, while significance (*P* < 0.05) of means was compared by the Student-Newman-Keuls (SNK) test. Pronounced reduction (*P* < 0.001) of cholesterol in overt as compared to control and subclinical was noticed, whereas marked improvement (*P* < 0.001) was evidenced in follow-up. Prominent elevation (*P* < 0.05) of TG in follow-up was evidenced as compared to control. Overt presented marked reduction of HDL-C as compared to subclinical and control (*P* < 0.01 and *P* < 0.001), respectively. Pronounced elevation (*P* < 0.001) of HDL-C was evidenced after treatment. Overt presented reduction of LDL-C as compared to subclinical and control (*P* < 0.01 and *P* < 0.05, respectively). The follow-up group demonstrated considerable (*P* < 0.001) improvement of LDL-C after treatment and elevation (*P* < 0.05) as compared to control. Overt presented reduction of Apo-B as compared to subclinical and control (*P* < 0.05 and *P* < 0.001, respectively). Improvement (*P* < 0.05) of Apo-B was evidenced in follow-up. Reduction (*P* < 0.05) of Apo-A in overt as compared to control and elevation (*P* < 0.05) in follow-up as compared to overt was evidenced. Conclusively, improvement after treatment was evidenced in lipid profile.

## 1. Introduction

Thyroid hormones both tri-iodothyronine (T_3_) and tetra-iodothyronine (T_4_) or thyroxin are the most vital biomolecules in the realm of thyroidology [1]. Both T_3_ and T_4_ are under the influence of thyroid-stimulating hormone (TSH) a glycoprotein, released by adenohypophysis that in turn is controlled by thyroliberin of the hypothalamus [2]. Levels of T_3_ and T_4_ in circulation execute a negative feedback effect on *pars distalis* of the pituitary to synthesize TSH, a regulatory hormone, which later acts on the TSH receptor (TSH-R) situated on the cellular membranes of thyroid follicles [3]. TSH mediates iodine uptake, primarily through the actions of sodium/iodide symporter [4].

Overt or clinical hyperthyroidism is characterized by elevated serum free T_4_ and T_3_ levels in the presence of an undetectable TSH concentration. Indications of hyperthyroidism are loss of weight, increased sweating, tremors of hands, weakness, increased appetite, accelerated heartbeat, loose stools, irritability, exophthalmos, heat intolerance, moist skin, and goiter [5].

Subclinical or asymptomatic thyroid disorder characterizes the initial phase of thyroid dysfunction [6]. It is attributed with elevated cardiac rate, enhanced left ventricular mass with marginal concentric changes, atrial arrhythmias, decreased ventricular relaxation phase, low exercise performance, and increased risk for cardiovascular expiry [7].

Thyroid hormones have been suggested to influence lipoprotein metabolism [8, 9]. High-density lipoprotein cholesterol (HDL-C), total cholesterol (TC), and triglycerides (TG) are major constituents of the lipid fraction of the human plasma [10].

Cholesterol, a waxy steroid metabolite, is a major constituent of human cellular membranes. Within the cellular milieu, cholesterol also functions in nerve conduction intracellular transport and cell signaling [11]. Perturbed thyroid hormone levels influence directly on the cholesterol metabolism as elevated level of cholesterol is an independent risk factor of coronary artery disease (CAD) [12]. Moreover, elevated level of low-density Lipoprotein cholesterol (LDL-C) is also considered to be a significant marker of abnormal cardiac health [13].

High-density lipoprotein particles are heterogeneous in their chemistry [14, 15] as more than 48 HDL-associated proteins are discovered, out of which 22 are linked with cholesterol and lipoprotein metabolism, while 23 are linked with acute phase reactant and the remaining 3 are involved in the complement activation [16]. A normal level of HDL particle not only reduces the chances of atherosclerotic events but also has an anti-inflammatory, antioxidative, and antiapoptotic role [17].

Triglycerides are esters formed by a combination of glycerol and fatty acids and are supposedly most prevalent and reliable energy reserves in the human body [10]. Increasing evidence suggests that elevated levels of TG with Apo-B are significant contributing factors in the progression of coronary heart disease (CHD) [18].

Dyslipidemia is a metabolic situation leading to a persistent increase in plasmatic level of triglycerides and cholesterol [19]. High levels of triglycerides (TG) and low levels of HDL cholesterol (HDL-C) are considered to be risk factors for CHD [20].

Apolipoproteins are the major constituents of lipoprotein particles, and there is an increasing trend that measurement of different forms of apolipoproteins may forecast the risk of cardiovascular events [21]. Raised concentration of apolipoprotein Apo-B, a cardinal constituent of atherogenic lipoproteins, and declined level of Apo A-I, a constituent of antiatherogenic HDL, are attributed to increased chances of MI [22, 23]. T_3_ is involved in the transcription of the apolipoproteins, depicting that altered thyroid state can affect the lipoprotein metabolism [24].

The present study is chalked out to evaluate the cardiovascular risks attributed with elevated levels of thyroid hormones and responsiveness of lipid biochemical parameters upon rehabilitating thyroid profile.

## 2. Materials and Methods

The present investigation is a part of Ph.D. dissertation “Predicitive Biomarkers of Cardiovascular Risks in Hyperthyroid Patients from Lahore, Pakistan,” submitted to University of the Punjab, Lahore.

For this prospective analysis, the Ethical Review Committee of institute of Zoology, University of the Punjab, approved this investigation. The study encompasses 179 age-matched subjects, recruited from tertiary care hospitals of Lahore, comprising both genders and fulfilling the inclusion and exclusion criteria. Healthy controls (*n* = 60; 40 females and 20 males), subclinical (*n* = 34; 21 females and 13 males), and overt hyperthyroid (*n* = 50; 35 females and 15 males) were securitized. Out of the 50 newly diagnosed hyperthyroid patients, a group (*n* = 35; 10 males and 25 females) agreed for follow-up study [25].

All of the subjects in the follow-up group were orally administered antithyroid drug Carbimazole. An average of the 30-60 mg of drug was administered orally per day; however, some patients with mild thyroid sickness were administered 10-20 mg/day. The quantity was reduced as the levels of FT_4_ and clinical symptoms were reduced [26]. Patients who underwent radioactive iodine treatment or thyroidectomy were not selected for the follow-up study.

Selected patients from all studied groups were monitored closely, and after careful final evaluation, they were subjected to biochemical evaluation.

As our investigation centered on human subjects, therefore, all health and precautionary measures were strictly observed. Research grade, genuine and sterilized, disposable syringes of B.D. (Becton Dickinson Pakistan, Private Limited) were used to draw 5 cc venous blood. Each of the participants was sampled for blood after having 12 hours of fasting.

Following blood was drawn, it was divided equally into two aliquots. One aliquot was specified for serum collection; there, it was allowed to clot at room temperature. After clot formation, it was centrifuged (Model NF 1215, Nuve, Turkey) at 3000 rpm for 5 minutes to separate serum.

The other aliquot was placed in EDTA-coated, precooled, and sterile vacuum blood collection tube (BOLTON). Plasma was collected by centrifuging the blood at 3000 rpm for 5 minutes and transferred into a sterilized labeled Eppendorf vial. Both serum and plasma were stored at -80°C. Further hormonal and biochemical assessment was performed within a week of phlebotomy.

Total cholesterol (TC), high-density lipoprotein-C (HDL-C), low-density lipoprotein-C (LDL-C), and triglycerides (TG) were measured by using commercially available diagnostic kits of DiaSys, Germany, whereas apolipoprotein-A (Apo-A) and apolipoprotein-B (Apo-B) were analyzed using research grade Glory Biosciences (ELISA) kits.

The quantitative determination was made by using clinical chemistry analyzer (Model 5010, Robert Riele GmbH & Co. KG, D-13467 Berlin, Germany) and ELISA workstation (ROBONIK absorbance microplate reader).

Assessment of thyroid profile, free-T_4_ (FT_4_), free-T_3_ (FT_3_), and thyroid-stimulating hormone (TSH), was done by Radioimmunoassay (RIA) with commercially available kits of Beckman Coulter of Czech Republic at Institute of Nuclear Medicine and Oncology Lahore (INMOL), Pakistan.

Informed permission in writing was collected from each of the participants of investigation. A detailed proforma was drafted to assess the demographic variables of the participated individuals. Exclusion criteria for subjects with thyroid disorder were cardiovascular complications, hypertension, diabetes mellitus, obesity, and family positive history of these ailments. Participants with positive history of hepatic impairment and drug addiction were not included in this investigation, as these situations may affect normal metabolism and physiology of the concerned individual. Pregnant women were excluded from this study.

Commercially available digital blood pressure equipment of CITIZEN (Micro HumanTech CH-452) was utilized to measure blood pressure (mmHg) of all enrolled participants. Weight (kg) and height (m) of all studied subjects were measured for assessment of body mass index (kg/m^2^). Average clothing weight was also subtracted from the measured body weight of all the participants.

Subjects having 11.5–23.0 pmol/L of free-T_4_, 5–5.8 pmol/L of free-T_3_, and 0.3–5 mIU/L of TSH were considered as normal.

### 2.1. Statistical Analysis

Statistically, biochemical comparisons among all groups, *viz*, control (C), subclinical (S), clinically overt hyperthyroid (O), and follow-up (F) were accomplished by applying one-way ANOVA [27]. As the treatments were having unequal numbers, hence, significant means (*P* < 0.05) were compared by the “Student-Newman-Keuls” (SNK) test.

## 3. Results

An overall representation of thyroid and lipid profile in comparable groups is presented in Table 1.

### 3.1. Total Cholesterol (TC)

**Figure 1:**
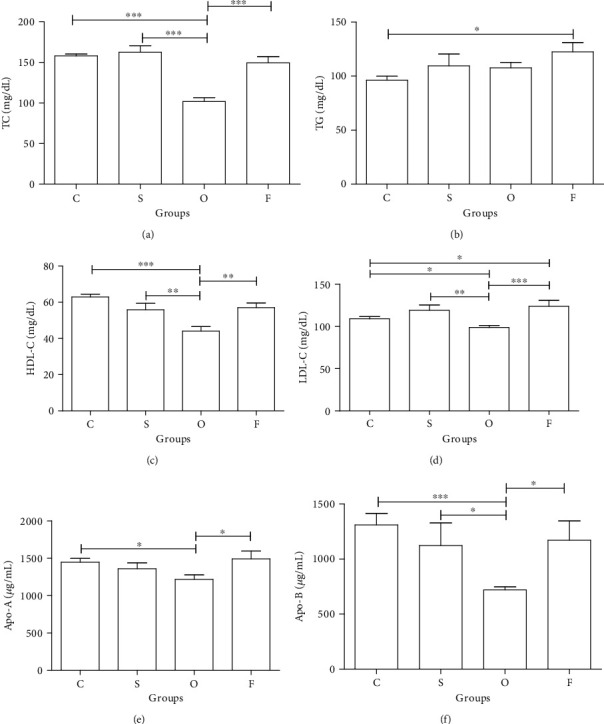
(a–f) Values are mean ± SEM. Significant at ^∗^*P* ≤ 0.05, ^∗∗^*P* ≤ 0.01, and ^∗∗∗^*P* ≤ 0.001.

Nonsignificant difference was present in control vs. subclinical and follow-up comparison; however, control vs. overt comparison presented marked difference (*P* ≤ 0.001) with 36% reduction of TC level in the overt group when compared with controls. Prominent difference (*P* ≤ 0.001) was noticed in subclinical vs. overt comparison with 37% reduction of TC in the overt group as compared to controls, while subclinical vs. follow-up comparison demonstrated nonsignificant difference. Lastly, marked difference (*P* ≤ 0.001) was observed with 47% increment of TC in overt vs. follow-up comparison, after antithyroid treatment (Figure 1(a)).

### 3.2. Triglycerides (TG)

Statistically nonsignificant difference was noticed in control vs. subclinical and overt comparison, while significant difference (*P* ≤ 0.05) was present in control vs. follow-up comparison with 27% elevation of TG in follow-up when compared with controls. Moreover, all other group comparison like subclinical vs. overt, subclinical vs. follow-up, and overt vs. follow-up demonstrated nonsignificant difference (Figure 1(b)).

### 3.3. High-Density Lipoprotein-C (HDL-C)

Statistically nonsignificant difference was noticed in the comparison of control vs. subclinical and follow-up, while highly significant difference (*P* ≤ 0.001) was present in the comparison of control vs. overt with 30% decrease of HDL-C in the overt group, when compared with controls. Twenty-one percent (significant at *P* ≤ 0.01) reduction of HDL-C was observed in the overt group, when compared to subclinical, while subclinical vs. follow-up comparison did not present significant difference. Lastly, marked difference (*P* ≤ 0.01) was observed in comparison of overt vs. follow-up with 29% elevation of HDL-C in the follow-up group, after antithyroid treatment (Figure 1(c)).

### 3.4. Low-Density Lipoproteins (LDL-C)

Statistically nonsignificant difference was noticed in comparison of control vs. subclinical, whereas control vs. overt comparison presented significant difference (*P* ≤ 0.05) with 9% decrement of LDL-C in the overt group. In control vs. follow-up scenario, statistically marked (*P* ≤ 0.05) difference was observed with 14% elevation of LDL-C in the follow-up group as compared with controls. Subclinical vs. overt comparison presented statistically significant difference (*P* ≤ 0.01) with 18% decrease of LDL-C in the overt group as compared to controls, while nonsignificant difference was observed in the comparison of subclinical vs. follow-up. In the last comparison of overt vs. follow-up, it demonstrated statistically highly significant difference (*P* ≤ 0.001) with 25% elevation in the follow-up group, after treatment (Figure 1(d)).

### 3.5. Apolipoprotein-A (Apo-A)

Nonsignificant difference was noticed in the group comparison of control vs. subclinical and follow-up, while significant difference (*P* ≤ 0.05) was observed in the comparison of control vs. overt with 16% decrease in the overt group, when compared with controls. Nonsignificant difference was observed in subclinical vs. overt and follow-up comparison. Lastly, the comparison of overt vs. follow-up presented significant difference (*P* ≤ 0.05) with 23% elevation of Apo-A in the follow-up group, after antithyroid treatment (Figure 1(e)).

### 3.6. Apolipoprotein-B (Apo-B)

Mild difference was noticed in the comparison of control vs. subclinical and follow-up, while highly significant difference (*P* ≤ 0.001) was present in the comparison of control vs. overt groups with 45% decline of Apo-B in the overt group, when compared with controls. Nonsignificant difference was observed in subclinical vs. follow-up comparison, while subclinical vs. overt comparison presented significant difference (*P* ≤ 0.05) with 36% decline in the overt group, when compared with the subclinical group. Lastly, in overt vs. follow-up comparison, significant difference (*P* ≤ 0.05) was observed with 62% increase in the follow-up group after antithyroid treatment (Figure 1(f)).

## 4. Discussion

Thyroid being a major endocrine gland with follicular cells [28] markedly affects lipid biosynthesis and its distribution. Lipids are organic compounds whose pivotal function is to provide energy. They are also main constituents of biomembrane, providing vital precursor for important hormones [29, 30].

In our findings, the concentration of cholesterol and LDL-C is reduced in hyperthyroid patients [31]. In subjects with reduced plasma cholesterol level, it is usually considered as an index of pathophysiological frailty, indicating the impending danger and it may also reflect incapability of the organism to meet with the cholesterol demand of body [32]. The trend of reduced cholesterol in hyperthyroid subjects can only be elucidated by its increased removal. Actually, TH upregulates the expression of cholesterol 7 *α*-hydroxylase, a prominent regulatory gene of the production of bile acids involved in the biliary secretion of bile acids and cholesterol, leading towards the decreased absorption of cholesterol in the intestine [33]. In addition to that, other proteins involved in the Reverse Transport of Cholesterol (RCT) mechanism, like ATP-binding cassette 1 and scavenger receptor B-I could be involved in increased removal of plasma cholesterol [34].

In clinical hyperthyroid condition, there is an enhanced hepatic expression of LDL-C receptors, which in turn are regulated by the T_3_-mediated LDL receptor gene. Furthermore, LDL receptor's gene expression is also indirectly regulated by T_3_ via sterol regulatory element-binding protein-2 (SREBP-2) [35].

Thyroid hormones regulate the production of 3-hydroxy-3-methylglutaryl-coenzyme A (HMG-CoA) reductase, which is the initial reaction in cholesterol biosynthesis machinery. It has been observed that thyroid hormone increases the protein, mRNA, and functioning of HMG-CoA reductase [33, 36].

Interestingly, hepatic lipogenesis is markedly amplified in hyperthyroid condition. This enhanced synthesis of lipids is also associated with increased lipolysis and its oxidation, thus, leading to their reduced levels in serum [37, 38].

The trend of enhanced lipogenesis is also observed in the animal model studies with excessive iodothyronines. Moreover, thyroid hormones can influence the homeostasis of the liver tissue by modifying its hormonal and biochemical conditions. Glucose and insulin both play a crucial role in the regulation and expression of vital genes in hepatic lipogenic and glycolytic cascades [39, 40]; though slight elevation in glucose and insulin concentrations of hyperthyroid patients could thus have played a role. This is all the more possible, because glucose has been shown to act in synergy with thyroid hormone to stimulate the expression of lipogenic genes [41, 42].

This simultaneous production and breakdown present increased energy demands and hypermetabolic activity in hyperthyroidism [43]. No evidence of decreased biosynthesis of cholesterol has yet been reported [44]. Conclusively, plasma low cholesterol in overt hyperthyroid condition is only due to increased clearance rate.

In the case of the subclinical hyperthyroid group, a slight increase of cholesterol and LDL-C was observed in our investigation [31]. As mentioned above, in overt hyperthyroid condition, lipogenesis is enhanced with increased clearance rate, so it is conceivable that during the subclinical phase of ailment, the elimination of cholesterol and LDL-C is not as fast as in the overt thyroid condition. This slight alteration in the lipid profile can forecast the tendency of atherosclerotic condition in subclinical hyperthyroidism [45]. Normalization of thyroid profile after having antithyroid treatment markedly influenced the level of cholesterol and LDL-C.

Moreover, in many clinical situations, gradual reversal of hypocholesterolemia is considered a marker of reversal of critical illness and of patient recovery; likewise, severe persistent hypocholesterolemia, or further extreme drop in cholesterol, is a strongly unfavourable prognostic sign [32]. It is worth mentioning here that decreased BMI observed in hyperthyroid condition can be an outcome of hypermetabolic condition and increased removal of lipids from the body.

The levels of triglycerides were mildly elevated in both the subclinical and overt groups [31]. This increasing trend can be explained from the fact that catabolism of triglycerides stored in adipose tissue is elevated by increased thyroid hormones; subsequently, it results in an increased concentration and turnover of nonesterified fatty acids (NEFA) [46]. Lipid oxidation rate is enhanced that results in increased availability of fatty acids [44, 47]. At hepatic level, this trend contributes to an elevated synthesis of ketone bodies. *In vivo* studies have shown increased TG levels in hyperthyroid subjects. This can only be justified by increased turnover rate [46] and delivery of NEFA to the liver, resulting in an elevated availability of fatty acyl-coenzyme A (acyl-CoA) for hepatic reesterification, as plasma NEFA delivery to liver parenchyma is a significant contributor to hepatic TG biosynthesis and secretion [48, 49].

The complicated relationship between coronary atherosclerosis and hypertriglyceridemia has been difficult to explain [50]. In addition to that, statistically, it is difficult to determine whether TG is a prominent risk factor for coronary heart disease (CHD). This may be due to its greater biological variability in TG levels than the cholesterol [51].

However, in many experimental, genetic, and epidemiological investigations, increased triglyceride levels, remnant cholesterol, and triglyceride-rich lipoproteins (TRLs) collectively, have demonstrated as important risk factors for cardiovascular diseases [52].

Interestingly, in greatly exceeded concentrations of TG, TRLs become too large to enter into the arterial intima and to manifest atherosclerosis. However, in comparison with mildly elevated triglyceride (TG) levels, the size of TRLs became small; thus, they enter easily into the arterial wall and accumulate to manifest atherosclerosis [53–55]. It is a well-established fact that conditions with reduced HDL-C, increased TG levels, and prothrombotic state can contribute to CAD [50].

Hence, moderately increased TG levels with reduced HDL-C in both subclinical and clinical hyperthyroid states can manifest CAD. Moreover, in the follow-up, the level of TG was increased. This may be due to reduced secretion of TG attaining a euthyroid state.

HDL-C has been known to have vital atheroprotective activities including anti-inflammatory, antioxidative, endothelial cell maintaining, and performing reverse cholesterol transport (RCT). Moreover, HDL-C is profoundly renowned as “good” cholesterol as high levels of HDL-C are attributed with decreased levels of cardiovascular events, and reduced concentration of HDL-C is associated with increased CVD, in many epidemiological investigations [56]. Navab and coresearchers pioneered cell-based and cell-free assays to measure the anti-inflammatory and antioxidant activities of HDL [57].

We have observed reduced concentration of HDL-C in the overt and subclinical hyperthyroid groups [31]. It is reported that hepatic lipase is enhanced in the clinical hyperthyroid situations; pertinently, this elevated hepatic lipase activity could pronouncedly enhance the catabolic pathway of the HDL particles [58].

Cholesteryl ester transfer protein (CETP) in plasma is increased in patients with hyperthyroidism and decreased in those with reduced thyroid function compared [13]. CETP is a hydrophobic glycoprotein that mediates the transfer of neutral lipids between HDL_2_ to the very low-density lipoproteins (VLDL), thus reducing their levels in the body; it also plays a cardinal role in the metabolism of HDL and Apo A-I and in the reverse cholesterol transport (RCT) pathway [35, 59]. So this prominent decline in the HDL-C could be due to the changes in HDL-C subfraction [60].

Moreover, it is an established fact that the elevated activity of hepatic lipase is reduced during the treatment of overt hyperthyroid sickness [58] so it is conceivable that after effective antithyroid treatment, the decreased level of HDL-C would be restored. The same trend of increasing HDL-C particles after antithyroid treatment is observed in our study.

Lipoproteins are one of the major biomolecules in the human milieu. Thyroid hormones have been suggested to influence lipoprotein metabolism [61]; of them, apolipoproteins are of vital importance [8]. Impairment of thyroid functions brings about pathological changes in different organs of the body [62].

This multimolecular complex is responsible for the transport of lipids between plasma and extracellular fluids. This large multigene family expression is regulated by a number of hormonal, developmental, and nutritional factors [63]. Apo-B is synthesized in enterocytes and hepatocytes as an obligate component of triglyceride-rich lipoproteins [64].

In our investigation, we observed a reduced level of Apo-B and Apo-A in subclinical as well as clinical groups [31]. In different studies, a reduced concentration of Apo-B has been reported [58, 65]. A decreased concentration of Apo-B can be explained from the fact that elevated thyroid hormone level may suppress the biosynthesis of Apo-B in the body.

Moreover, in an animal model study of pharmacologically induced hypothyroid rats, an increased level of stop codon in the Apo-B_100_ mRNA has been reported upon the administration of T_3_, suggesting that TH may have a modulatory effect in the induction of stop codon in the mRNA of Apo-B_100_, resulting in the reduced presence of Apo-B in the plasma [66]. Normalization of the thyroid profile also exerted a pronounced improvement in the Apo-B levels.

Usually, a nonatherogenic lipoprotein profile is evidenced in patients with clinical hyperthyroidism before and also after treatment [67].

In our investigation, reduced levels of Apo-A have been observed in clinical hyperthyroid subjects [31]. These finding are in accordance with the previous findings of Diekman et al. [65] and Minarikova et al. [67]. Factors that influence the chemistry and size of the HDL lipid are also supposed to modulate Apo A-I metabolism [13].

Apolipoprotein A-I level tends to mirror the changes in HDL-cholesterol. HDL particles can also be classified by the apolipoprotein contribution [68]. Apo A-I and A-II are the salient contributing proteins present in HDL and constitute the two important HDL subclasses: those that contain only Apo A-I (LpA-I) and those that are having both Apo A-I and Apo A-II (LpA-I:A-II). Reduced concentrations of LpA-I have been reported in previous investigations, as it has Apo-A1 in its moiety, so it is understandable that declined Apo-A1 low level may be due to LpA-I low levels. Apo-AI influences indirectly the size of HDL particle because enhanced catabolism of Apo-AI manifests a pronounced decline in HDL particle [13]. In the follow-up group, an improvement in the Apo-AI level is observed suggesting that Apo-AI levels in the clinical hyperthyroid conditions are of revisable in nature.

## 5. Conclusion

Conclusively, improved lipid biosynthesis has been observed in the follow-up group attaining a euthyroid state. Moreover, subjects of hyperthyroidism usually indicate normal levels of lipid profile. However, the physicians should regularly recommend the follow-up of lipid profile, as mildly elevated triglyceride levels with TRLs do have the potential to manifest cardiac abnormalities.

## 6. Limitations of the Study

The study has been conducted in Lahore city, Punjab province, Pakistan. However, a detailed investigation is suggested in other provinces and cultures of the country to have a better picture of this ailment.

## Figures and Tables

**Table 1:** A comprehensive presentation of thyroid and lipid profile in studied groups.

Parameters	Control	Subclinical	Overt	Follow-up	*P* value
FT_4_ (pmol/L)	17.53 ± 0.36	19.36 ± 0.93	45.65 ± 2.49	14.66 ± 1.08	<0.0001^∗∗∗^
FT_3_ (pmol/L)	3.91 ± 0.09	4.62 ± 0.20	28.77 ± 1.94	4.96 ± 0.16	<0.0001^∗∗∗^
TSH (mIU/L)	1.97 ± 0.15	0.09 ± 0.01	0.04 ± 0.007	0.72 ± 0.214	<0.0001^∗∗∗^
TC (mg/dL)	157.9 ± 2.39	162.5 ± 8.01	101.8 ± 4.84	149.5 ± 7.53	<0.0001^∗∗∗^
TG (mg/dL)	96.18 ± 3.69	109.6 ± 10.85	87.00 ± 3.57	122.4 ± 8.62	0.033^∗^
HDL-C (mg/dL)	62.88 ± 1.56	55.85 ± 3.48	44.00 ± 2.57	57.00 ± 2.57	<0.0001^∗∗∗^
LDL-C (mg/dL)	109.0 ± 2.64	119.1 ± 6.35	98.68 ± 2.51	123.8 ± 7.12	0.0002^∗∗∗^
Apo-B (*μ*g/mL)	1310 ± 104.4	1122 ± 206.1	720.4 ± 28.18	1171 ± 97.28	0.0002^∗∗∗^
Apo-A (*μ*g/mL)	1448 ± 50.74	1360 ± 79.76	1218 ± 58.87	1493 ± 102.8	0.0164^∗^

Values are means ± SEM. ∗ indicates significance at *P* < 0.001. FT_4_: free tetraiodothyronine; FT_3_: free tri-iodothyronine; TSH: thyroid-stimulating hormone; TC: total cholesterol; TG: triglycerides; HDL-C: high-density lipoprotein-C; LDL-C: low-density lipoprotein-C; Apo-B: apolipoprotein-B; Apo-A: apolipoprotein-A.

## Data Availability

The data used to support the findings of this study are included within the article.
