# Corrigendum to “Prospective Analysis of Lipid Variations in Hyperthyroid Subjects from Lahore, Pakistan”

**DOI:** 10.1155/2022/9815891

**Published:** 2022-01-06

**Authors:** Muhammad Amir Iqbal, Shaaf Ahmad, Tamseela Mumtaz, Zahra Naseem, Javeria Malik, Husna Ahmad, Nabila Roohi

**Affiliations:** ^1^Institute of Zoology, University of the Punjab, Canal Road, Lahore, Punjab 54590, Pakistan; ^2^King Edward Medical University/Mayo Hospital, Hospital Road, Lahore, Punjab 54000, Pakistan; ^3^Department of Zoology, Government College for Women University, Faisalabad, Punjab, Pakistan

In the article titled “Prospective Analysis of Lipid Variations in Hyperthyroid Subjects from Lahore, Pakistan” [1], the authors have identified minor errors in Figure 1 and Table 1. The corrected Figure 1 and Table 1 are as follows:

## Figures and Tables

**Figure 1:**
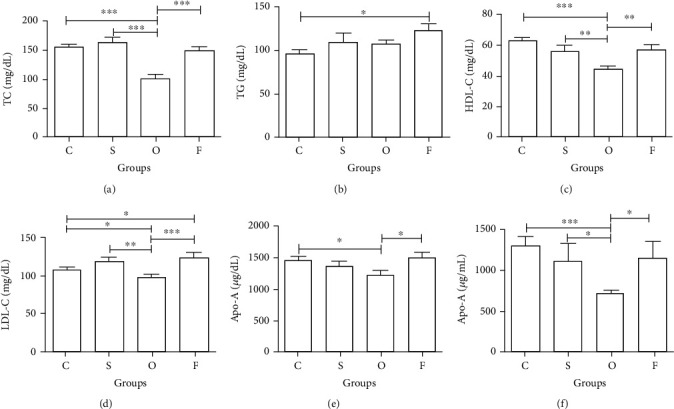
Comparison of the levels (mean ± SEM) of different study parameters (a–f) in control (C), subclinical (S), overt (O), and follow-up (F) groups showing significant differences at ^∗^*P* ≤ 0.05, ^∗∗^*P* ≤ 0.01, and ^∗∗∗^*P* ≤ 0.001.

**Table 1:** A comprehensive presentation of thyroid and lipid profile in studied groups.

Parameters	Control	Subclinical	Overt	Follow-up	*P*-value
FT_4_ (pmol/L)	17.53 ± 0.36	19.36 ± 0.93	45.65 ± 2.49	14.66 ± 1.08	<0.0001
FT_3_ (pmol/L)	3.91 ± 0.09	4.62 ± 0.20	28.77 ± 1.94	4.96 ± 0.16	<0.0001
TSH (mIU/L)	1.97 ± 0.15	0.09 ± 0.01	0.04 ± 0.007	0.72 ± 0.214	<0.0001
TC (mg/dL)	157.9 ± 2.39	162.5 ± 8.01	101.8 ± 4.84	149.5 ± 7.53	<0.0001
TG (mg/dL)	96.18 ± 3.69	109.6 ± 10.85	87.00 ± 3.57	122.4 ± 8.62	0.03
HDL-C (mg/dL)	62.88 ± 1.56	55.85 ± 3.48	44.00 ± 2.57	57.00 ± 2.57	<0.0001
LDL-C (mg/dL)	109.0 ± 2.64	119.1 ± 6.35	98.68 ± 2.51	123.8 ± 7.12	0.0002
Apo-B (*μ*g/mL)	1310 ± 104.4	1122 ± 206.1	720.4 ± 28.18	1171 ± 97.28	0.0002
Apo-A (*μ*g/mL)	1448 ± 50.74	1360 ± 79.76	1218 ± 58.87	1493 ± 102.8	0.0164

Values are means ± SEM. FT_4_: free tetra-iodothyronine; FT_3_: free tri-iodothyronine; TSH: thyroid-stimulating hormone; TC: total cholesterol; TG: trigylcerides; HDL-C: high-density lipoprotein-C; LDL-C: low-density lipoprotein-C; Apo-B: apolipoprotein-B; Apo-A: apolipoprotein-A.
